# Loss of *Sphingosine Kinase 1*/S1P Signaling Impairs Cell Growth and Survival of Neurons and Progenitor Cells in the Developing Sensory Ganglia

**DOI:** 10.1371/journal.pone.0027150

**Published:** 2011-11-09

**Authors:** Hui Meng, Yuan Yuan, Vivian M. Lee

**Affiliations:** 1 Division of Developmental Biology, Department of Pediatrics, Medical College of Wisconsin, Milwaukee, Wisconsin, United States of America; 2 Tumor Etiology and Screening Department of Cancer Institute and General Surgery, The First Affiliated Hospital of China Medical University, Shenyang, China; City of Hope National Medical Center and Beckman Research Institute, United States of America

## Abstract

**Background:**

Lysophospholipids such as lysophosphatidic acid (LPA) and sphingosine-1-phosphate (S1P) are important signaling molecules that can regulate a wide range of cellular responses. We discovered that *Sphingosine kinase 1* (*Sphk1*), a key enzyme that converts sphingosine to S1P, is expressed in neurons and progenitor cells in nascent trigeminal and dorsal root ganglia during mouse embryogenesis.

**Methods and Findings:**

*Sphk1* null mouse embryos do not display overt deficits owing to compensation by *Sphk2*. Thus, we analyzed embryos that are deficient in both *Sphk1* and *Sphk2* (which essentially eliminates S1P function) in order to investigate the role(s) of *Sphk1* during sensory ganglia formation. While animals lacking 1–3 alleles of *Sphk1* and *Sphk2* had no obvious phenotype, embryos without both genes displayed clear developmental defects. The complete absence of *Sphk1* and *Sphk2* resulted in trigeminal and dorsal root ganglia with fewer neurons and progenitor cells. The profound loss in cell number could be attributed to a decrease in cell proliferation as well as an increase in apoptosis. Furthermore, *Sphk1*/*2* double mutants displayed an overall reduction in other sphingolipids as well as an imbalance of S1P/sphingosine and S1P/ceramide ratio, thereby favoring cell death and reducing cell growth.

**Conclusions:**

Together, these results provide strong in vivo evidence that sphingosine kinase/S1P signaling plays an important role in regulating early events during development of sensory ganglia.

## Introduction

Sensory neurons convey senses, among others, of touch, temperature, and proprioception from the periphery to the central nervous system. The sensory system is made up of the cranial ganglia (which include the trigeminal ganglia and dorsal root ganglia). Both neurons and glia in the dorsal root ganglion are derived from the multipotent neural crest cells and the trigeminal ganglion receives additional neuronal contribution from the trigeminal placode in the head [1]. As sensory neurons differentiate and innervate target sites, their survival is supported by neurotrophic factors such as the family of neurotrophins [2], [3], [4], [5]. On the other hand, what regulate progenitor cell proliferation and neuronal survival during early ganglia development is less well understood. Ciliary neurotrophic factor and neurotrophin-3 have been demonstrated to induce proliferation of progenitor cells and neuronal differentiation in immature dorsal root ganglia [6]. We recently discovered that *Sphingosine kinase 1* was expressed in progenitor cells and a subset of neurons in the nascent sensory ganglia and hypothesized that the Sphingosine kinase/sphingosine-1-phosphate could mediate cell proliferation and survival prior to target innervation.

Lysophospholipids such as lysophosphatidic acid (LPA) and sphingosine-1-phosphate (S1P) are membrane-derived bioactive lipid mediators [7], [8], [9]. S1P, its precursor, sphingosine, and ceramide are sphingolipids that have been studied by many groups as they are involved in signaling pathways that control multiple cellular processes including cell proliferation, differentiation, survival and migration ([8], [9], [10], [11]. Sphingosine is phosphorylated by Sphingosine kinases to form S1P [12] and sphingosine can be generated either by degradation of sphingolipids or by deacylation of ceramide. S1P can be dephosphorylated by *Sphingosine-1-phosphate phosphatases 1* and *2* (*Sgpp1* and *Sgpp2*) or by lipid phosphohydrolases with broader substrate specificity. Alternatively, S1P can be irreversibly degraded to ethanolamine phosphate and hexadecenal (long chain aldehyde) by the action of sphingosine-1-phosphate lyase.

Sphingolipids regulate cell growth and survival in many cell types. S1P is usually associated with cell proliferation and survival; in contrast, ceramide and sphingosine are usually associated with cell death [11], [12], [13], [14]. Stressful stimuli or growth factor withdrawal can activate sphingomyelinase, which converts sphingomyelin to produce ceramide. Ceramide can take part in multiple events that lead to stress response, growth arrest, or apoptosis. The enzyme ceramidase can further metabolize ceramide to produce sphingosine, which can inhibit protein kinase C and induce apoptosis. Thus, the relative levels of S1P, ceramide, and sphingosine inside a cell can influence survival or apoptosis [13], [14], [15]. These sphingolipid molecules are interconvertible, higher level of S1P favors cell growth, whereas cell death ensues when the reaction tends towards sphingosine/ceramide; thus, this balance is sometimes referred to the “sphingolipid rheostat” [16], [17], [18]. Of all the molecules that participate in the sphingolipid rheostat, *Sphingosine kinase* is particularly important because it can decrease ceramide and sphingosine by catalyzing the formation of S1P. In mammals, two sphingosine kinases, *Sphingosine kinase 1 (Sphk1)* and *Sphingosine kinsae 2 (Sphk2)* have been identified. The biological function of *Sphk2* is controversial and less well characterized than *Sphk1*. *Sphk1* is upregulated in several types of cancers, including cancer of the colon, breast, stomach, and kidney [18]. Additional evidence also suggested that tumor cells can overexpress *Sphk1*, implicating the S1P pathway in the uncontrolled growth and dampening of cell death associated with cancers [18].

Many actions of S1P are mediated by G protein coupled receptors. In mammals there are at least five S1P receptors (*S1pr1-5*) [19]. Some of the S1P receptors are expressed in the nervous system [20], [21] and play a role in the development of neurons and glia cells. *S1pr1*, for example, is necessary for brain development [22] and the survival of oligodendrocyte precursors requires the activity of *S1pr5* [23]. However, S1P can also act as a second messenger inside cells and elicit various cellular responses [24], [25]. In addition to mobilizing intracellular Ca^2+^ [24], recent studies reported that intracellular S1P could regulate histone deacetylases and the ubiquitin ligase activity of tumor necrosis factor receptor-associated factor 2 [10], [26], [27]. Here, we demonstrate a role for the S1P pathway in sensory ganglion development and show that complete loss of *Sphk* activity leads to severe disruption of neuronal survival and proliferation of progenitor cells in the developing trigeminal and dorsal root ganglia.

## Materials and Methods

### Ethics statement

All studies using mice were conducted according to Institutional Animal Care and Use Committee (IACUC) guidelines of the Medical College of Wisconsin with approval, protocol AUA198. Mice were housed and maintained in the Bioresource Center of the Medical College of Wisconsin, which is accredited by the Association for the Assessment and Accreditation of Laboratory Animal Care (AAALAC).

### Animals and genotyping

*Sphk1*, *Sphk2*, *S1pr1*, and *S1pr2* mutant mice were generated and characterized as described [22], [28], [29], [30]. Double mutant mice were generated by intercrossing *Sphk1−/−;Sphk2+/−* mice. Genotyping by PCR was carried out as described [20].

### Whole mount in situ hybridizations

E9.5 (22–25 somites) and E10.5 (32–35 somites) wild type, *Sphk1−/−; Sphk2+/− Sphk1−/−; Sphk2−/−* embryos were fixed in 4% paraformaldehyde overnight at 4°C, rinsed in phosphate buffered saline, and dehydrated in methanol series and stored in 100% methanol at −20°C. Embryos were processed for whole mount in situ hybridization as previously described [31] and color reaction developed with BM Purple (Roche). Whole mount and section in situ hybridizations for each probe were repeated at least three times to confirm our results.

### Section in situ hybridization and immunfluorescence

For section in situ hybridizations, E10.5 (32–35 somites) and E11.5 (40–42 somites) embryos were fixed with modified Carnoy's solution, processed for paraffin sectioning (10 µm), and in situ hybridization as described [32]. For post-in situ immunohistochemistry, slides were incubated in Tuj-1 or SOX10 overnight at 4°C [33]. Fluorochrome conjugated secondary antibodies (Jackson ImmunoResearch) were applied for 2 hours at room temperature in dark. Brightfield in situ hybridization and fluorescence immunostaining signals on sections were photographed using a Zeiss Z1 microscope equipped with a monochrome MRm AxioCam camera. Brightfield signals were inverted in Adobe Photoshop (from purple brightfield signal to green) and overlayed with Tuj-1 and SOX10 fluorescence signals. For immunofluorescence, wild type, *Sphk1−/−; Sphk2+/− Sphk1−/−; Sphk2−/−* embryos were embedded in gelatin, frozen in liquid nitrogen, and stored in −80°C. Serial cryosections (10 µm) were cut from head and trunk (between forelimb and hindlimb) regions of embryos and adjacent sections were collected on two sets of slides. One set of slides was stained for islet-1/2 and active-caspase 3 (act-Casp-3) and the second set of slides was stained for SOX10 and phospho-histone H3 (PH3).

### Cell counting and statistics

All sections including trigeminal ganglia and dorsal root ganglia (the first three DRG at the fore limb level) were photographed and Tuj-1+, active-caspase 3+ (from one set of slides) and SOX10+, phospho-histone H3+ (a second set of slides with alternate sections) cells were counted using ImageJ. One trigeminal ganglion and two DRG from each embryo were counted, and three embryos for each group were used in the analyses. Data are expressed as means ± standard error of means. Student's t-test was used for statistics, *p* values of p<0.01 or p<0.05 were considered significant.

### Measurement of S1P, sphingosine and ceramide levels

Three E11.5 (40–42 somites) embryos from wild type, *Sphk1−/−; Sphk2+/−, and Sphk1−/−; Sphk2−/−* group were collected and frozen immediately at −80°C. S1P, sphingosine, and ceramide analyses were performed by the Lipidomics core of Medical University of South Carolina. Data are expressed as means ± standard error of means. Student's t-test was used for statistics, p values of p<0.01 or p<0.05 were considered significant.

## Results

### *Sphingosine kinase 1* is expressed in the developing trigeminal and dorsal root ganglia

We have previously shown that the developing sensory ganglia express very low levels of S1P receptors during early gangliogenesis ([20] and data not shown). To investigate the potential role of another key component in the S1P pathway, *Sphingosine kinase 1* (*Sphk1*), in the developing sensory ganglia, we first examined its expression in these structures. We performed section in situ hybridization on trigeminal and dorsal root ganglia sections in E10.5 (32–35 somites) and E11.5 (40–42 somites) embryos.

**Figure 1:**
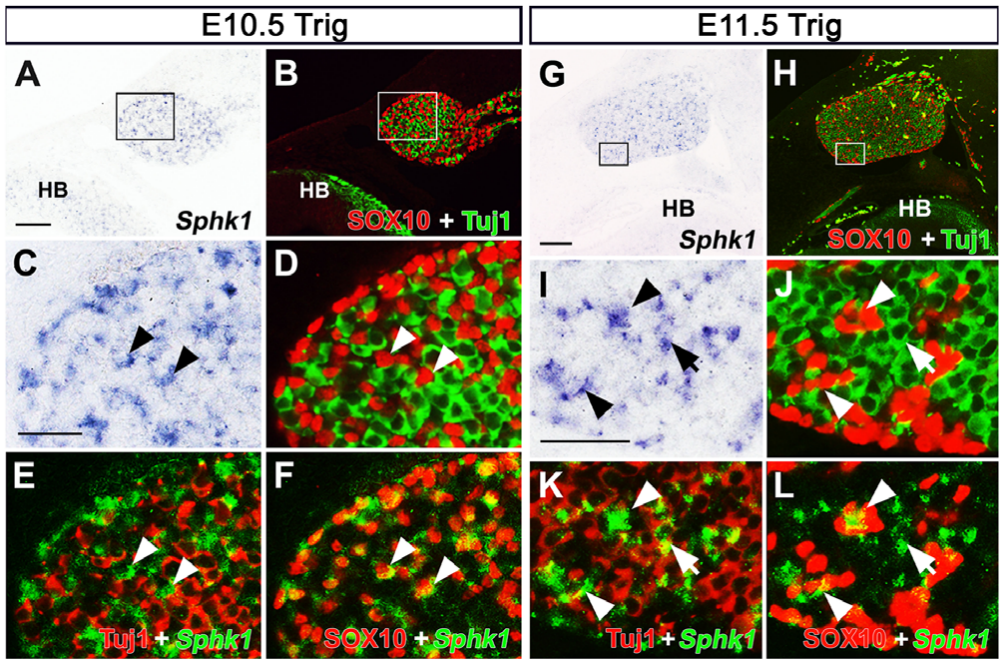
*Sphk1* expression in the developing trigeminal ganglion. *Sphk1* transcript was detected by section in situ hybridization followed by Tuj1 and SOX10, immunostaining. (**A–F**) *Sphk1* expression in E10.5 (32–35 somites) trigeminal ganglion. (**A**) *Sphk1* mRNA **(purple)** can be observed in the trigeminal ganglion and hindbrain (HB). (**B**) The same section in (**A**) was processed for Tuj-1 (green) and SOX10 (red) immunostaining. (**C–F**) Enlarged image of boxed area in A and B. (**C**) *Sphk1* (purple) is not expressed in all cells in the trigeminal ganglion. (**D–F**) Co-staining with Tuj-1 (green) and SOX10 (red) reveals that *Sphk1* can be seen in Sox10+ progenitor cells (arrowhead) but not Tuj1+ neurons at E10.5. (**E, F**), *Sphk1* signal was pseudo-colored as green, Tuj1 and SOX10 immunostaining as red. *Sphk1* signals are not found in Tuj-1+ neurons **(arrowhead)**. *Sphk1* signal (green) co-localized with SOX10+ cells **(arrowhead)**. (**G–L**) *Sphk1* expression in E11.5 (40–42 somites) trigeminal ganglion. (**G**) *Sphk1* **(purple)** expression persists in E11.5 trigeminal ganglion. (**H**) The same section in (**G**) was processed for Tuj-1 **(green)** and SOX10 **(red)** immunostaining. (**I–L**) Enlarged imaged of boxed area in G and H. (I, J) *Sphk1* **(purple)** is expressed in a subpopulation of cells in the trigeminal ganglion. *Sphk1* can be observed in SOX10+ cells **(arrowhead)**. Some neurons **(Tuj1+, green)** also express *Sphk1* at this stage **(arrow)**. (**K,L**) *Sphk1* in situ signal was pseudo-colored green, Tuj1 and SOX10 reactivity as red to observe overlap of in situ and immuostaining signals. *Sphk1* mRNA **(green)** can be detected in some neurons at E11.5 trigeminal ganglion **(arrow)**. A subset of SOX10+ cells express *Sphk1* **(arrowhead)**. **HB** = hindbrain. **Scalebar** for A, B = 200 µm; **scalebar** for C–F = 50 µm; **scalebar** for G, H = 100 µm; **scalebar** for I–L = 50 µm.

At E10.5, *Sphk1* transcript was scattered throughout the trigeminal ganglion but was not expressed by all the cells (Fig. 1A, B). To identify which cell types expressed *Sphk1*, we co-labeled the same section with Tuj-1 and SOX10 to identify neurons and neural crest progenitor cells, respectively. By examining higher magnification of single labeled and merged images, we found that *Sphk1* was expressed in many SOX10+ cells but not Tuj-1+ cells at this stage (Fig. 1C–F). At E11.5, *Sphk1* expression pattern appeared similar to E10.5. Co-labeling experiments showed that *Sphk1* continued to be observed in SOX10+ cells but now some neurons also began to express the *Sphk1* mRNA at E11.5 (Fig. 1G–L).

**Figure 2:**
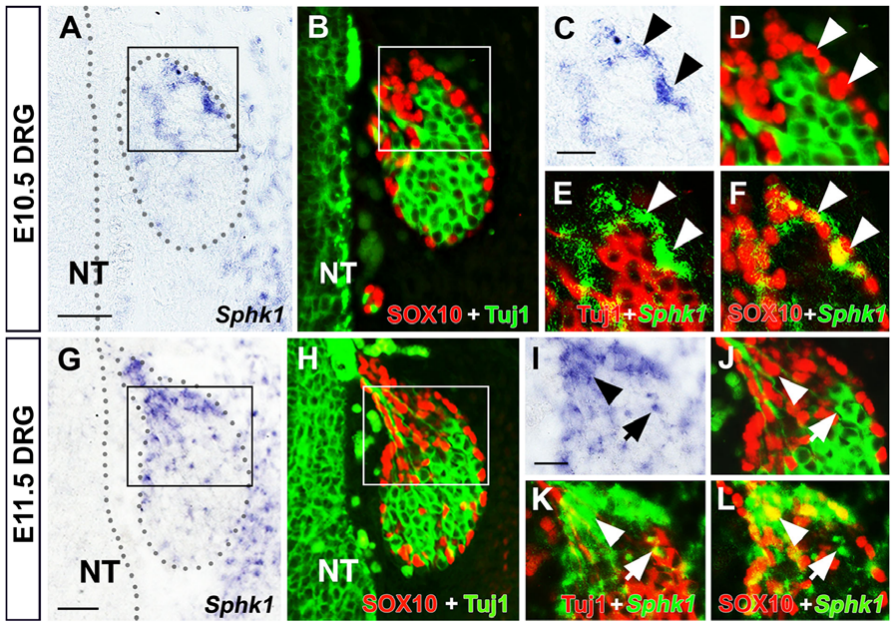
*Sphk1* expression in the developing dorsal root ganglion. *Sphk1* transcript was detected by section in situ hybridization followed by Tuj1 and SOX10 immunostaining. (**A–F**) *Sphk1* expression in E10.5 (32–35 somites) dorsal root ganglion. (**A**) *Sphk1* in situ signal (purple) can be detected in some cells in the dorsal root ganglion at this stage **(DRG outlined by grey dots)**. (**B**) The same section in A processed for Tuj-1 **(red)** and SOX10 **(green)** immunofluorescence. Most of the SOX10+ neural crest progenitor cells are found at the periphery and cap region of the ganglion while Tuj1+ neurons are more centrally located. (**C–F**) Enlarged image of boxed area in A and B. (**C,D**) *Sphk1* transcript is primarily located in the periphery of the DRG and co-incides with the occurrence of SOX10+ cells **(arrowhead)**. (**E,F**) In these panels, *Sphk1* in situ signal is pseudo-colored green, Tuj-1 (red, E) and SOX10 (red, **F**) to visualize co-localization. *Sphk1* mRNA is associated with SOX10+ cells **(arrowheads)**; Tuj1+ neurons do not express *Sphk1* at this time. (**G–L**) *Sphk1* expression in E11.5 (40–42 somites) dorsal root ganglion. (**G**) *Sphk1* signal **(purple)** is seen mostly in the cap region of the ganglion **(DRG outlined by grey dots)**. (**H**) Same section in **G** immunostained with Tuj1 **(green)** and SOX10 **(red)**. Majority of the SOX10+ cells **(red)** are located in the periphery and cap region of the DRG but several SOX10+ cells can also be found in the central region. (**I–L**) Enlarged image of boxed area in G and H. (**I,J**) Similar to E10.5, many SOX10+ cells express *Sphk1* **(arrowhead)** but some neurons **(arrow)** also express *Sphk1* at E11.5. (**K,L**) *Sphk1* signal is pseudo-colored green and Tuj1 red (K), SOX10 red (**L**) to confirm co-localization of signals. *Sphk1* mRNA is found in SOX10+ cells **(arrowhead)**, cells that are *Sphk1*+/SOX10+ appear yellow in **L**. Some neurons express *Sphk1* at this time **(arrow)**. **NT** = neural tube. **Scalebar** for A, B and G, H = 50 µm; **scalebar** for C–F and I–L = 20 µm.

At E10.5, we detected *Sphk1* in the periphery of the dorsal root ganglia. Immunostaining with SOX10 in the same section confirmed that *Sphk1* co-localized with some SOX10+ cells, suggesting that progenitor cells expressed *Sphk1*. We did not detect *Sphk1* transcripts in DRG neurons (Tuj-1+) at this stage (Fig. 2A–F). At E11.5, we observed *Sphk1* in the periphery of the DRG and some scattered *Sphk1*+ cells were present throughout the ganglion. Co-labeling experiments showed that *Sphk1* was expressed in both SOX10+ progenitors as well as Tuj-1+ neurons at this time (Fig. 2G–L).

### Depleting Sphingosine kinase activity causes defects in sensory ganglia

To investigate the possible roles of *Sphk1* in the developing nervous system, we aimed to ablate *Sphk1* in the mouse embryo. In mammals, there are two Sphingosine kinases, *Sphk1* and *Sphk2*, and knockout mice for both have been generated [20], [22]. *Sphk1−/−* or *Sphk2−/−* single mutants do not show any obvious phenotype, survive until adulthood, and are fertile [22], [28]. Previous studies demonstrated that *Sphk2* mRNA levels were not altered in *Sphk1* null animals compare to controls. Furthermore, S1P levels were found to be similar or just slightly decreased in *Sphk1−/−* tissues compared to wild type [28]. As we do not observe any sensory ganglia defects in the *Sphk1* single mutant (Fig. S2), *Sphk2* may be able to compensate for the absence of Sphk1 by maintaining sufficient S1P albeit it is expressed at low levels in sensory ganglia (Fig. S1).

**Figure 3:**
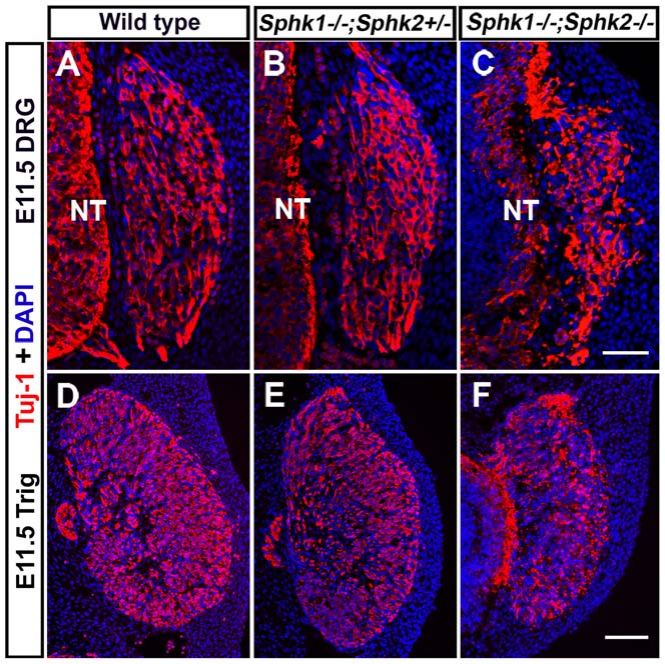
Sensory ganglia in *Sphk1−/−;Sphk2−/−* embryos appear disorganized and smaller. Head and trunk transverse sections from E11.5 (40–42 somites) wild type, *Sphk1−/−;Sphk2+/−*, and *Sphk1−/−;Sphk2−/−* mice were processed for Tuj1 **(red)** immunofluorescence and counterstained with DAPI **(blue)** to examine morphologies of the trigeminal **(Trig)** and dorsal root ganglia **(DRG)**. (**A–C**) Trunk sections showing E11.5 DRG in wild type, *Sphk1−/−;Sphk2+/−*, and *Sphk1−/−;Sphk2−/−* embryos. (**A,B**) DRG from wild type and *Sphk1−/−;Sphk2+/−* are similar in shape, size, and Tuj1+ neurons look normal. (**C**) In contrast, *Sphk1−/−;Sphk2−/−* DRG has a less well-defined shape and there appear to be fewer neurons. (**D–F**) Sections showing trigeminal ganglia from wild type, *Sphk1−/−;Sphk2+/−*, and *Sphk1−/−;Sphk2−/−* embryos. (**D,E**) Trigeminal ganglia from wild type and *Sphk1−/−;Sphk2+/−* are similar in shape, size, and contain many Tuj1+ neurons. (**F**) *Sphk1−/−;Sphk2−/−* trigeminal ganglion is smaller and may have fewer neurons. **NT** = neural tube. **Scalebar** for A–C = 50 µm; **scalebar** for D–F = 100 µm.

In contrast to *Sphk1* or *Sphk2* single mutant, *Sphk1/2* double knockout mice have severe phenotypes [22], [28]. Deleting both *Sphk1* and *Sphk2* eliminates S1P function, and because S1P is important for vascular development, *Sphk1−/−;Sphk2−/−* embryos die at E12.5 due to vascular defects [22]. We generated *Sphk1−/−;Sphk2−/−* double mutant embryos in order to examine the role of sphingosine kinase activity in sensory ganglia development. Similar published results, we observed that *Sphk1−/−;Sphk2−/−* double mutant mice die at E12-12.5 and the embryos are slightly smaller than their littermates and wild type embryos [22]. We examined trigeminal and dorsal root ganglia in *Sphk1−/−;Sphk2−/−* double mutant embryos at E11.5 (40–42 somites) and compared them to controls. In wild type and *Sphk1−/−;Sphk2+/−* animals, trigeminal and dorsal root ganglia were formed properly and the morphology of neurons was normal, as demonstrated by Tuj-1 immuostaining. In contrast, *Sphk1−/−;Sphk2−/−* sensory ganglia appeared smaller and disorganized (Fig. 3A–F). There appeared to be fewer Tuj-1+ neurons in both trigeminal and dorsal root ganglia in *Sphk1−/−;Sphk2−/−* embryos. These defects will be analyzed in more details and presented in the following sections of the manuscript.

**Figure 4:**
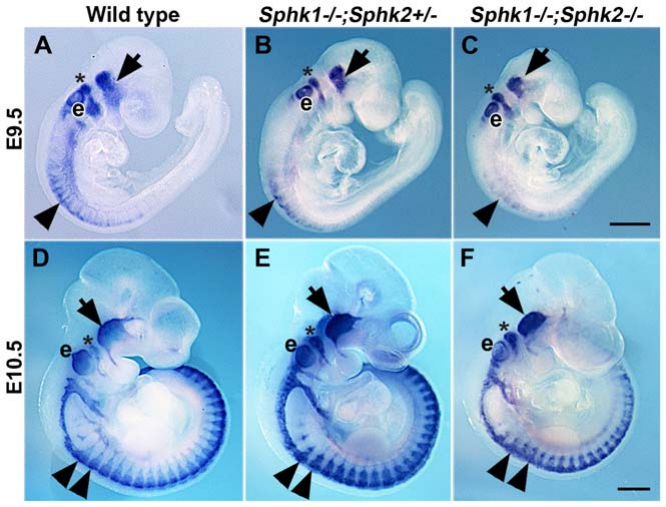
Deleting *Sphk1* and *Sphk2* does not alter neural crest migration pattern. *Sox10* whole mount in situ hybridization was performed on wild type, *Sphk1−/−;Sphk2+/−*, and *Sphk1−/−;Sphk2−/−* embryos at E9.5 (22–25 somites) and E10.5 (32–35 somites). (**A–C**) At E9.5, *Sox10* expression can be detected in the forming trigeminal ganglia **(arrow)**, hindbrain neural crest **(asterisk)** as well as the developing ear (**e**). Trunk neural crest cells are beginning to migrate in the first few somites **(arrowhead)**. Embryos from all three genotypes show similar *Sox10* staining pattern even though the *Sphk1−/−;Sphk2−/−* embryo is slightly smaller (**C**). (**D–F**) At E10.5, trunk neural crest migration **(arrowhead)** is well underway and can be visualized by *Sox10* staining. Trigeminal ganglia **(arrow)** and hindbrain neural crest cells **(asterisk)** can be identified at this stage. *Sox10+* neural crest cells are present in the *Sphk1−/−;Sphk2−/−* embryo and they display the stereotypical segmental migration pattern similar to the wild type embryo although the stream of neural crest cells in *Sphk1−/−;Sphk2−/−* double mutant embryo appears narrower comparing to wild type and *Sphk1−/−;Sphk2+/−* embryos. The ear (**e**) continues to express *Sox10*. **Scalebars** for A–C and D–F = 500 µm.

Neural crest stem cells migrate and form trigeminal and dorsal root ganglia [1]. To dissect the basis of sensory ganglia dysgenesis in *Sphk1−/−;Sphk2−/−* animals, we first compared the migration pattern of neural crest cells in control and *Sphk1−/−;Sphk2−/−* embryos using *Sox10* whole mount in situ hybridization. E9.5 (22–25 somitets) and E10.5 (32–35 somites) wild type, *Sphk1−/−;Sphk2+/−* and *Sphk1−/−;Sphk2−/−* embryos were processed and compared together. At E9.5, the forming trigeminal ganglion and hindbrain neural crest cells can be visualized in the head. In the trunk, neural crest cells are beginning to migrate at the level of the first few somites. Migrating neural crest cells and trigeminal ganglia could be observed in E9.5 embryo in all three genotypes (Fig. 4A–C). Although the *Sphk1−/−;Sphk2−/−* embryos were slightly smaller, both cranial and trunk neural crest cells showed normal migratory patterns. By E10.5, the trigeminal ganglion is clearly formed and most of the trunk neural crest cells are migrating. The pattern of migration of neural crest cells did not seem to be altered in *Sphk1−/−; Sphk2−/−*; however, the streams of migrating cells appeared to be thinner compare to controls (Fig. 4D–F). Thus, neural crest formation and migration occurred in *Sphk1−/−;Sphk2−/−* mutant embryos and the defects observed in the sensory ganglia might take place shortly after their formation.

### Ablation of Sphingosine kinases disrupted trigeminal ganglion development

**Figure 5:**
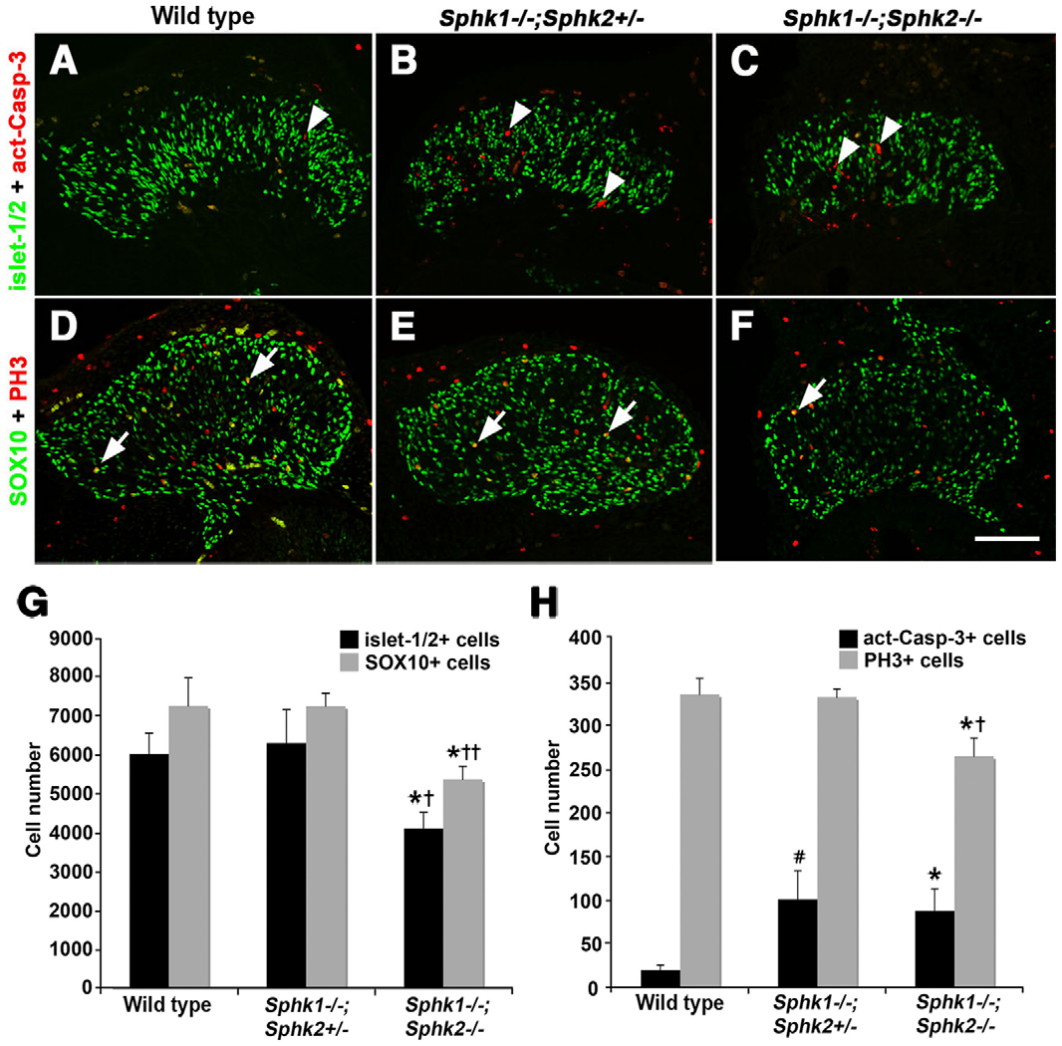
Loss of neurons and progenitor cells can be observed in E10.5 trigeminal ganglion in the *Sphk1−/−;Sphk2−/−* double mutant. (**A–C**) Trigeminal ganglion sections from wild type, *Sphk1−/−;Sphk2+/−*, and *Sphk1−/−;Sphk2−/−* were processed for activated Caspase 3 **(red, act-Casp-3)** and islet-1/2 **(green, nuclear)** immunofluorescence to identify dying cells and neurons, respectively. There are very few act-Casp-3+ cells in the wild type (A) trigeminal ganglion at E10.5 (32–35 somites). In comparison, both *Sphk1−/−;Sphk2+/−* (**B**), and *Sphk1−/−;Sphk2−/−* (**C**) trigeminal ganglia have a small but significant increase in act-Casp-3+ cells **(arrowhead in B,C; black bars in H)**. *Sphk1−/−;Sphk2−/−* trigeminal ganglia are smaller and contain fewer islet-1/2 positive neurons compare to wild type and *Sphk1−/−;Sphk2+/−* ganglia (**A–C; black bars in G**). (**D–F**) Adjacent sections from those used in (**A–C**) were immunostained for phosphoshistone H3 **(PH3, red)** and SOX10 **(green, nuclear)** to identify dividing cells and neural crest progenitors, respectively. (**D–F**) PH3+ cells can be found throughout the trigeminal ganglia in all three genotypes examined and some PH3+ are dually positive for SOX10 **(white arrowheads in D–F)**. There are fewer PH3+ and SOX10+ cells in *Sphk1−/−;Sphk2−/−* trigeminal ganglion (**F; grey bars in G,H**). (**G**) Quantification of the total number of **islet-1/2+ (neurons, black bars)** and **SOX10+** **(progenitor cells, grey bars)** cells reveals that while the number of neurons and progenitor cells are similar in E10.5 wild type and *Sphk1−/−;Sphk2+/−* trigeminal ganglia, both islet-1/2+ and SOX10+ cells are significantly reduced in *Sphk1−/−;Sphk2−/−*. n = 3 embryos from each genotype; * = *Sphk1−/−;Sphk2−/−* to wild type comparison, p<0.05; **†** = *Sphk1−/−;Sphk2−/−* to *Sphk1−/−;Sphk2+/−* comparison, p<0.05; **††** = *Sphk1−/−;Sphk2−/−* to *Sphk1−/−;Sphk2+/−* comparison, p<0.01. (**H**) Quantification of the total dying **(act-Casp-3+, black bars)** and dividing **(PH3+, grey bars)** cells confirms that there is very little cell death in E10.5 wild type trigeminal ganglion. A small but significant increase in the number of dying cells can be seen in *Sphk1−/−;Sphk2+/−* and *Sphk1−/−;Sphk2−/−* double mutant compared to the wild type. n = 3 embryos from each genotype; * = *Sphk1−/−; Sphk2−/−* to wild type comparison, p<0.05; **^#^** = *Sphk1−/−;Sphk2+/−* to wild type comparison, p<0.05; **†** = *Sphk1−/−;Sphk2−/−* compare with *Sphk1−/−;Sphk2+/−*, p<0.05. **Scalebar** for A–F = 100 µm.

Because *Sphk1−/−;Sphk2−/−* trigeminal ganglia appeared smaller and might contain fewer cells at E10.5 (Fig. 3D–F), we carried out a series of double immunostaining experiments at this embryonic stage and quantitatively studied different cell populations, together with cell death, and cell proliferation assays. First, we assessed cell death/apoptosis. We used anti-activated caspase 3+ (act-Casp3) and anti-Islet-1/2 antibodies to determine the number of dying cells and neurons, respectively. In wild type ganglia, there were a small number of act-Casp3+ cells as expected due to naturally occurring cell death at this age. When we compared the number of dying cells in wild type, *Sphk1−/−;Sphk2+/−*, and *Sphk1−/−;Sphk2−/−* trigeminal ganglia, there was a slight increase in cell death in the *Sphk1−/−;Sphk2+/−* and double mutants (Fig. 5A–C, H, Table 1). Next, we assessed cell proliferation. We examined the number of mitotic cells and progenitor cells by using phosphohistone H3 (PH3) and SOX10 as markers. We found that there were 334±18 dividing cells in wild type and 331±9 in *Sphk1−/−;Sphk2+/−*, while *Sphk1−/−;Sphk2−/−* trigeminal ganglia only contained 263±21 PH3+ cells (Fig. 5D–F). Some of the PH3+ cells also co-labeled with SOX10 (Fig. 5D, E). Although the number of PH3+ cells was lower (263±21) in *Sphk1−/−;Sphk2−/−* compared to controls (∼331), the percentage of dividing cells compared to the total number of cells in the ganglia was essentially the same amongst these genotypes (∼2.5% on average, Table 1).

**Table 1:** Quantification of cell numbers in control and *Sphk1−/−;Sphk2−/−* sensory ganglia.

	E10.5 Trigeminal ganglia			
	Wild type	*Sphk1−/−; Sphk2+/−*	*Sphk1−/−; Sphk2−/−*	% Reduction
Neurons (islet-1+)	6018±535	6302±858	4113±414	32
precursors (SOX10+)	7252±718	7245±330	5373±330	26
total cells	13270±1242	13547±1188	9485±744	29
Active-caspase3+	19±6	100±33	87±25	
PH3+	334±18	331±9	263±21	
% of dying cells	0.1	0.7	0.9	
% of mitotic cells	2.5	2.4	2.8	

	E11.5 Trigeminal ganglia			
	Wild type	*Sphk1−/−; Sphk2+/−*	*Sphk1−/−; Sphk2−/−*	% Reduction
Neurons (islet-1+)	9435±411	10148±719	5531±887	41
precursors (SOX10+)	9464±513	9607±244	6463±664	32
total cells	18899±924	19755±963	11994±1551	37
Active-caspase3+	61±14	152±19	2343±369	
PH3+	397±11	402±34	17±14	
% of dying cells	0.3	0.8	19.5	
% of mitotic cells	2.1	2	0.1	

	E10.5 Dorsal root ganglia			
	Wild type	*Sphk1−/−; Sphk2+/−*	*Sphk1−/−; Sphk2−/−*	% Reduction
Neurons (islet-1+)	754±75	762±40	328±7	56
precursors (SOX10+)	500±57	423±21	379±28	24
total cells	1254±131	1185±28	707±26	44
Active-caspase3+	0.67±0.20	1.00±0.70	0.17±0.20	
PH3+	28±2	26±2	25±3	
% of dying cells	0	0	0	
% of mitotic cells	2.2	2.2	3.5	

	E11.5 Dorsal root ganglia			
	Wild type	*Sphk1−/−; Sphk2+/−*	*Sphk1−/−; Sphk2−/−*	% Reduction
Neurons (islet-1+)	1137±88	1058±25	770±24	32
precursors (SOX10+)	818±42	795±47	544±20	33
total cells	1955±85	1853±64	1315±43	33
Active-caspase3+	9±5	17±7	156±31	
PH3+	26±6	36±5	4±5	
% of dying cells	0.4	0.9	11.9	
% of mitotic cells	1.3	1.9	0.3	

To determine if depleting *Sphingosine kinases* affected both neurons and progenitor cells, we counted the total number of islet-1/2+ neurons and SOX10+ progenitors cells in the trigeminal ganglia at E10.5. In wild type and *Sphk1−/−;Sphk2+/−* trigeminal ganglia, there were 6,018 and 6,302 Islet-1/2+ neurons, respectively; whereas *Sphk1−/−;Sphk2−/−* ganglia had 4,113 neurons—a 32% decrease compared to the other two genotypes (Fig. 5A, G, Table 1). We also observed a significant decrease (26%) in the progenitor population. *Sphk1/2* double mutant ganglia had only 5,373 SOX10+ cells compared to 7,252 in wild type and 7,245 in *Sphk1−/−;Sphk2+/−*. However, because neither cell proliferation nor cell death was greatly affected in the double mutant trigeminal ganglia at E10.5, it is possible that the reduced number of neurons and progenitors was caused by a decrease in neuronal differentiation and/or a scarcity of progenitor cells.

**Figure 6:**
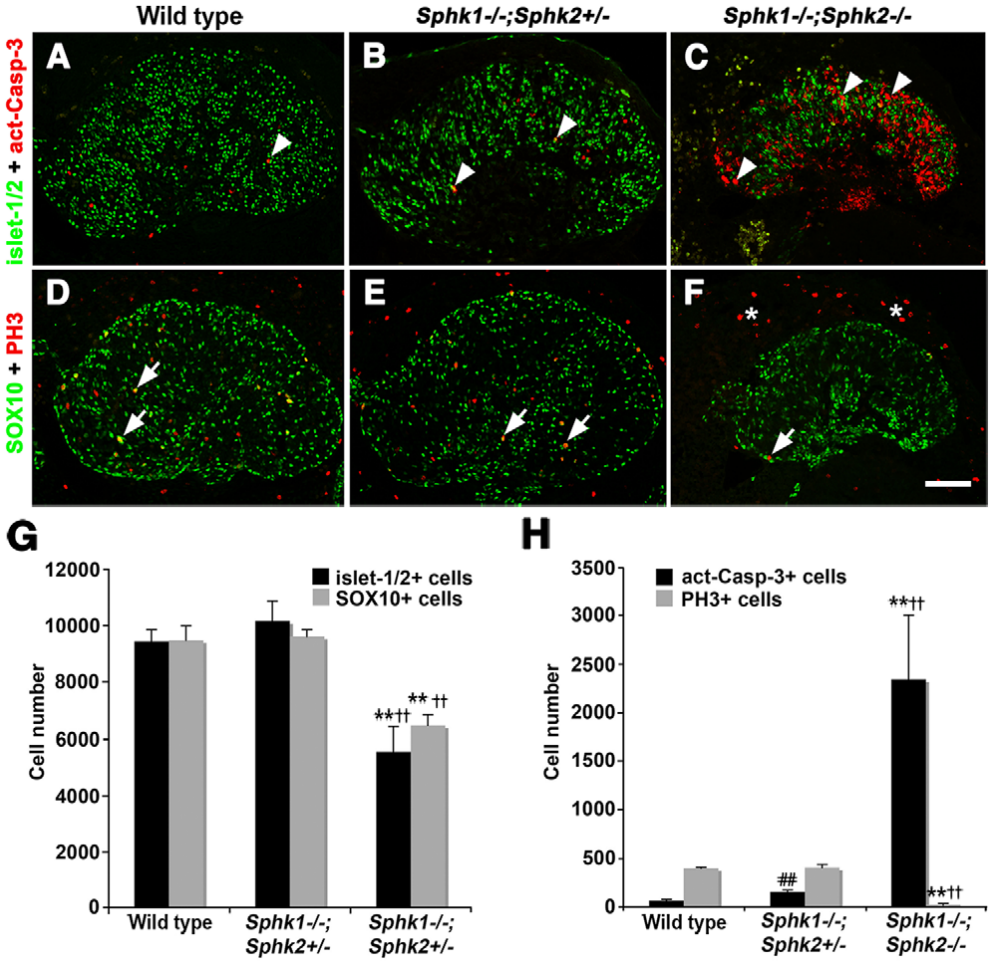
Cell loss is further exacerbated in E11.5 *Sphk1−/−;Sphk2−/−* trigeminal ganglion. (**A–C**) E11.5 (40–42 somites) trigeminal ganglia from wild type, *Sphk1−/−;Sphk2+/−*, and *Sphk1−/−;Sphk2−/−* were immunostained with antibodies to act-Casp-3 **(red, arrowhead)** and islet-1/2 **(green, nuclear)**. A small number of act-Casp-3+ cells can be seen in wild type and *Sphk1−/−;Sphk2+/−* ganglia; in contrast, there are many more act-Casp-3+ cells in *Sphk1−/−;Sphk2−/−* trigeminal ganglion. (**D–F**) Adjacent sections to those in (**A–C**) were immunostained with antibodies to PH3 **(red, arrow)** and SOX10 **(green, nuclear)**. PH3+ cells are scattered throughout the trigeminal ganglia from wild type (**D**) and *Sphk1−/−;Sphk2+/−* (**E**) embryos. (**F**) There are very few PH3+ cells in the *Sphk1−/−;Sphk2−/−* trigeminal ganglia; normal number of PH3+ cells outside the trigeminal ganglion were detected in the double mutant **(asterisks)**. (**G**) Quantification of total cell number shows that the number of neurons (islet-1/2+) and progenitor cells **(SOX10+)** are similar in the wild type and *Sphk1−/−;Sphk2+/−* at E11.5. There are significant decreases in neurons and progenitor cells in *Sphk1−/−;Sphk2−/−* double mutant ganglia. n = 3 embryos from each genotype; ** = *Sphk1−/−;Sphk2−/−* to wild type comparison, p<0.01. **^††^** = *Sphk1−/−;Sphk2−/−* to *Sphk1−/−;Sphk2+/−* comparison, p<0.01. (**H**) Compare to wild type, the increase of act-Casp-3+ dying cells is small and represents less than 1% of the total cell population in the *Sphk1−/−;Sphk2+/−* trigeminal ganglia, the number of dying cells increases to 2,343, almost 20% of the total cells in *Sphk1−/−;Sphk2−/−* double mutant trigeminal ganglia. We observe significant decrease of PH3+ dividing cells in the double mutant compare to wild type and *Sphk1−/−;Sphk2+/−* trigeminal ganglia at this stage. ** = *Sphk1−/−;Sphk2−/−* to wild type comparison, p<0.01. **^††^** = *Sphk1−/−;Sphk2−/−* to *Sphk1−/−;Sphk2+/−* comparison, p<0.01; **^##^** = *Sphk1−/−;Sphk2+/−* to wild type comparison, p<0.01. **Scalebar** for A–F = 100 µm.

Survival of trigeminal ganglion cells was compromised by the loss of *Sphk* activity as an increase in cell death was observed at E11.5. We performed cell death and proliferation analyses on E11.5 trigeminal ganglia and quantified cell numbers. In wild type and *Sphk1−/−;Sphk2+/−* trigeminal ganglia, only 0.3–0.8% (61–152 act-casp3+ cells out of 18,899–19,755 total cells) of all cells were dying; on the other hand, there were significantly more act-casp3+ cells throughout the *Sphk1−/−;Sphk2−/−* double mutant ganglia (2343±369, 19.5% of total cells; Table 1, Fig. 6A–C, H). In addition, the number of dividing cells was greatly reduced in *Sphk1−/−;Sphk2−/−* compared to control embryos (Fig. 6D–F, H). In *Sphk1/Sphk2* double mutants, there were only 17±4 PH3+ cells compared to 397±11 in wild type and 402±34 in *Sphk1−/−;Sphk2+/−* trigeminal ganglia. When we counted the total number of neurons and progenitor cells, we found that wild type and *Sphk1−/−;Sphk2+/−* trigeminal ganglia had an average of 10,000 neurons whereas *Sphk1−/−;Sphk2−/−* had about 5,531 (Table 1). The number of SOX10+ progenitors was also significantly lower. While wild type and *Sphk1−/−;Sphk2+/−* ganglia had an average of 9,500 SOX10+ cells, the double mutant only had ∼6,463 such cells. Thus, both neurons and non-neuronal cells showed a 35–40% loss in cell number when *Sphk1* and *Sphk2* were deleted. Survival of both neurons and progenitor cells were affected by the absence of *Sphks* as act-casp3+ cells were found in both islet-1/2+ and SOX10+ populations (Fig. S3).

### Deleting Sphks negatively influenced dorsal root ganglion development

**Figure 7:**
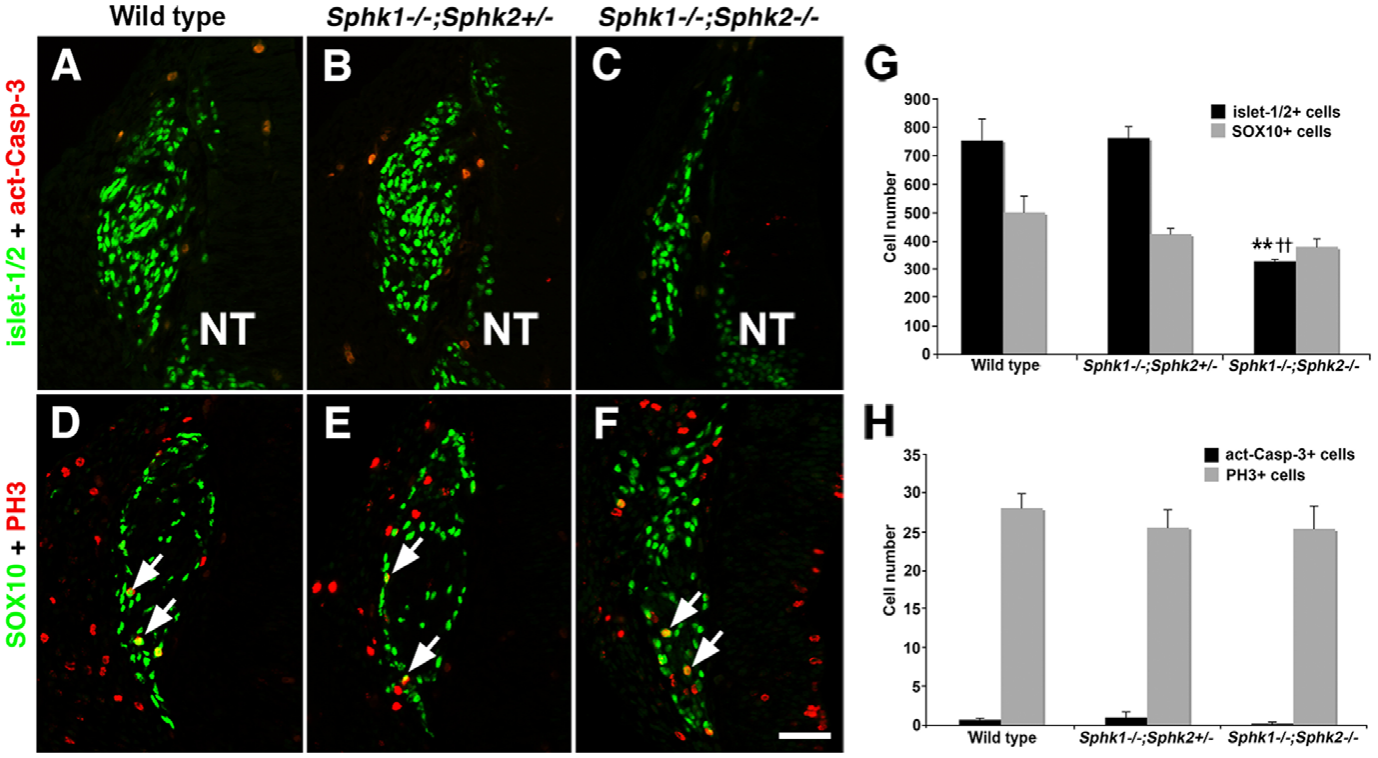
Ablation of *Sphk1* and *Sphk2* affects neuron but not progenitor cell numbers in E10.5 dorsal root ganglion. (**A–C**) Transverse trunk sections from wild type, *Sphk1−/−;Sphk2+/−*, and *Sphk1−/−;Sphk2−/−* immunostained with islet-1/2 **(green, nuclear)** and act-Casp-3 **(red)**. Wild type (**A**) and *Sphk1−/−;Sphk2+/−* (**B**) DRG appear similar in shape and size. *Sphk1−/−;Sphk2−/−* DRG (**C**) is smaller and has fewer neurons. Few act-Casp-3+ cells are observed in DRG from all three genotypes. (**D–F**) Adjacent sections to those used in (**A–C**) were processed for PH3 **(red)** and SOX10 **(green, nuclear)** immunofluorescence. The shapes of the DRG are similar in wild type (**D**) and *Sphk1−/−;Sphk2+/−* (**E**); most SOX10+ cells are located in the periphery of the ganglion. Sox10+ cells in *Sphk1−/−;Sphk2−/−* DRG are no longer restricted to the periphery of the ganglion. PH3+ **(green)** cells can be found in DRG from all genotypes examined and some of them are also SOX10 positive **(arrow)**. (**G**) Quantification of cell number in E10.5 DRG demonstrates that there are 24% fewer SOX10+ cells in *Sphk1−/−;Sphk2−/−* DRG compared to wild type and *Sphk1−/−;Sphk2+/−* but this reduction is not statistically significant (p = 0.079). The double mutant DRG shows a 56% reduction in the number of neurons compared to wild type and *Sphk1−/−;Sphk2+/−* embryos. n = 3 embryos from each genotype; **  = *Sphk1−/−;Sphk2−/−* to wild type comparison, p <0.01. **^††^**  = *Sphk1−/−;Sphk2−/−* to *Sphk1−/−;Sphk2+/−* comparison, p<0.01. (**H**) Quantification of total number of dying **(act-Casp-3+)** and dividing **(PH3+)** cells. Cell count results reveal no significant difference in cell death and proliferation in *Sphk1−/−;Sphk2−/−* DRG compared to wild type and *Sphk1−/−;Sphk2+/−* at this stage. **NT** = neural tube. **Scalebar** for A–F =  50 µm.

Next we examined if the spinal sensory ganglia defects were similar to those observed in the trigeminal ganglia. At E10.5, there were few act-Casp3+ cells (Fig. 7A–C, H) and only several PH3+ cells (Fig. 7D–F, H) in dorsal root ganglia from all genotypes. Although the number of SOX10+ cells in *Sphk1−/−;Sphk2−/−* was slightly lower compared to wild type and *Sphk1−/−;Sphk2+/−* dorsal root ganglia, the difference was not significant. Some PH3+ were dually positive for SOX10 (Fig. 7D–F, G). Even without major changes in cell death and proliferation, the double mutant DRG were still significantly smaller in size and there appeared to be fewer islet-1/2+ cells. Indeed when we counted the neurons, we found that there were ∼328 islet-1/2+ neurons in the double mutant ganglia compared to an average of ∼760 in controls (Fig. 7A–C, G; Table 1). Thus, the double mutant DRG had less than half the number of neurons compare to controls, suggesting that the lack of *Sphk* activity negatively influenced neuronal differentiation or survival or both of these events during the initial formation of the DRG.

**Figure 8:**
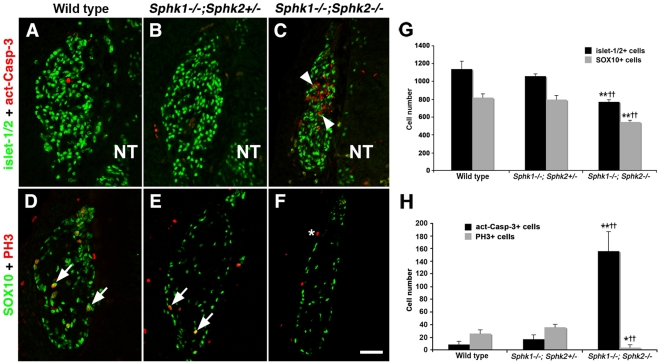
Cell loss in *Sphk1−/−;Sphk2−/−* becomes apparent in the dorsal root ganglia by E11.5. (**A–C**) E11.5 DRG section from wild type, *Sphk1−/−;Sphk2+/−*, and *Sphk1−/−;Sphk2−/−* embryos immunostained with antibodies to act-Casp-3 **(red)** and islet-1/2 **(green, nuclear)**. Very few act-Casp-3+ cells can be observed in wild type (**A**) and *Sphk1−/−;Sphk2+/−* (**B**) while *Sphk1−/−;Sphk2−/−* (**C**) shows significant cell death **(arrowhead)**. (**D–F**) Adjacent sections to those in (**A–C**) were stained with anti-PH3 **(red)** and SOX10 **(green)**. Both wild type (**D**) and *Sphk1−/−;Sphk2+/−* (**E**) DRG display PH3+ cells and some of them are also SOX10+ **(arrow)**. *Sphk1−/−;Sphk2−/−* DRG (**F**) is smaller and has fewer PH3+ cells **(red)**. (**G**) Quantification of total cell number shows that *Sphk1−/−;Sphk2−/−* DRG have significant loss (>30%) in both neurons **(black bars)** and progenitor cells compare to wild type and *Sphk1−/−;Sphk2+/−*. n = 3 embryos from each genotype; ** = *Sphk1−/−; Sphk2−/−* to wild type comparison, p<0.01. **^††^** = *Sphk1−/−; Sphk2−/−* to *Sphk1−/−;Sphk2+/−* comparison, p<0.01. (**H**) Wild type and *Sphk1−/−;Sphk2+/−* DRG have fewer than 20 act-Casp-3+ dying cells on average but *Sphk1−/−;Sphk2−/−* double mutants have an average of 156 dying cells. Cell proliferation is modest in E11.5 wild type and *Sphk1−/−;Sphk2+/−* DRG but is further reduced in the double mutant. n = 3 embryos from each genotype; * = *Sphk1−/−;Sphk2−/−* compare with wild type, p<0.05; ** = *Sphk1−/−;Sphk2−/−* to wild type comparison, p<0.01. **^††^** = *Sphk1−/−;Sphk2−/−* to *Sphk1−/−;Sphk2+/−* comparison, p<0.01. **NT** = neural tube. **Scalebar** for A–F = 50 µm.

By E11.5, cell death and proliferation were affected in *Sphk1−/−;Sphk2−/−* mutant DRG. At this stage, there were only a small number of dying cells in wild type and *Sphk1−/−;Sphk2+/−* ganglia, as normal cell death has not commenced [34]. On the other hand, many more act-Casp3+ cells were detected in the double mutant DRG (156±31; Table 1; Fig. 8A–C, H). Quantification showed that E11.5 *Sphk1−/−;Sphk2−/−* dorsal root ganglia had ∼156 act-Casp3+ compared to 9 in wild type and 17 in *Sphk1−/−;Sphk2+/−* embryos. Mitotic activity in normal dorsal root ganglia was modest at E11.5. We saw 26–36 PH3+ in wild type and *Sphk1−/−;Sphk2+/−* DRG and some PH3+ were also SOX10+. In the double mutant ganglia, even fewer PH3+ could be detected (4±5 PH3+ on average; Table 1; Fig. 8D–F, H).

Furthermore, both neurons and progenitor cells were depleted in the absence of *Sphks*. Wild type and *Sphk1−/−;Sphk2+/−* DRG had an average of ∼1,100 neurons while *Sphk1−/−;Sphk2−/−* double mutant ganglia had ∼770, representing a 32% loss in neurons. The decrease in SOX10+ cells was about ∼33% in the double mutant (544 SOX10+ cells) compared to controls (818 and 795 SOX10+ cells in wild type and *Sphk1−/−;Sphk2+/−* embryos, respectively).

### Deficits in sensory ganglia development can be attributed to the loss of Sphingosine kinase activity

**Figure 9:**
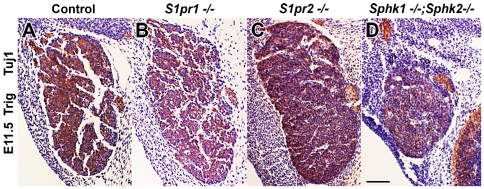
Trigeminal ganglia develop normally in *S1pr1* and *S1pr2* mutant mice. E11.5 (40–42 somites) trigeminal ganglia were sectioned processed for Tuj-1 immunostaining **(brown)**, hematoxylin was used as a counterstain **(blue)**. (**A–C**) Trigeminal ganglia from control, *S1pr1−/−* and *S1pr2−/−* appeared similar in size and contain many Tuj-1+ neurons. (**D**) A section from *Sphk1−/−;Sphk2−/−* trigeminal ganglion was included here for comparison. It was much smaller compared to *S1pr1−/−* and *S1pr2−/−* trigeminal ganglia. **Scalebar** for A–D = 100 µm.

Many effects of the S1P pathway are mediated through S1P receptors. In the *Sphk1−/−;Sphk2−/−* embryos, cell death and proliferation were affected in the brain at E11.5. The developing brain expresses abundant *S1pr1* [20], [22], and cell death and proliferation defects were obvious at E11.5 in *S1pr1* null animals [20]. These data suggested that perhaps S1P, acting through *S1pr1*, was at least partially responsible for CNS development. Besides the receptor-mediated activities of S1P, our data support a role for the intracellular activity of SPHK in regulating sensory ganglia development. To begin to address this issue, we compared trigeminal and dorsal root ganglia from *S1pr1* and *S1pr2* mutant mice [29], [30], [35] and *Sphk1;Sphk2* double knockout embryos. While *S1pr2−/−* mouse do not display overt neural phenotypes until three to seven weeks of age [30], *S1pr1−/−* embryos die between E12.5–E14.5 due to embryonic hemorrhage [29]. In contrast to *Sphk1/2* double mutants trigeminal and dorsal ganglia from E11.5, which were much reduced in size and cell number, *S1pr1* and *S1pr2* null sensory ganglia were similar to their control littermates (Fig. 9 and data not shown).

**Figure 10:**
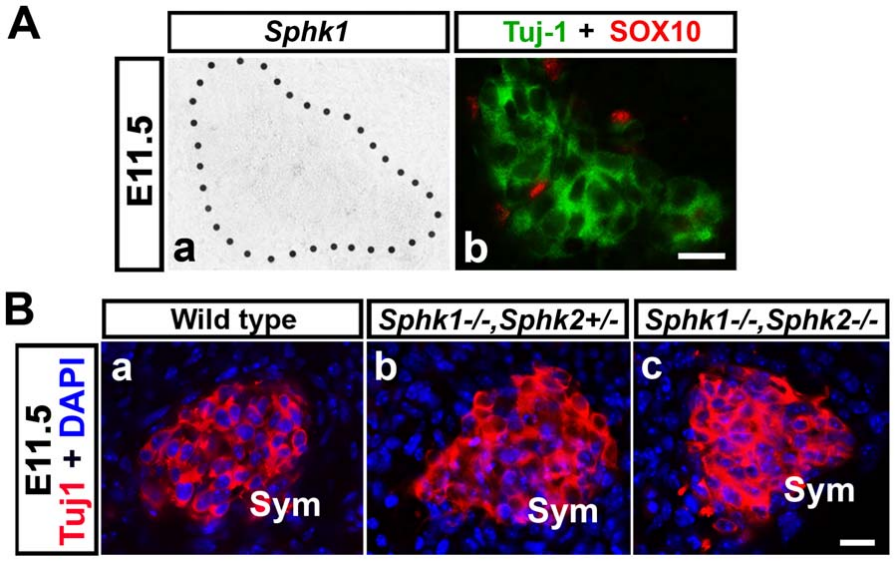
E11.5 sympathetic ganglia express little to no *Sphk1* and are not affected by the absence of *Sphk1* and *Sphk2*. (**A**) E11.5 (40–42 somites) mouse transverse sections including sympathetic ganglia were processed for *Sphk1* in situ hybridization followed by Tuj1 **(green)** and SOX10 **(red)** immunostaining. (**A-a**) Little *Sphk1* transcript can be detected in E11.5 sympathetic ganglion **(outlined by black dots)**. (**A-b**) Same section in (**A-a**) labeled with Tuj1 **(green)** and SOX10 **(red)**. (**B**) Transverse trunk sections from wild type, *Sphk1−/−;Sphk2+/−*, and *Sphk1−/−;Sphk2−/−* processed for Tuj1 immunofluorescence **(red)**, all nuclei were counterstained with DAPI **(blue)**. **(a–c)** Sympathetic ganglia from all three genotypes do not display any significant difference in size and morphology. **Sym** = sympathetic ganglia. **Scalebar** for A-a,b = 30 µm. **Scalebar** for B-a,b = 30 µm.

To investigate if the effects of ablating *Sphks* were specific to sensory ganglia, we examined the sympathetic ganglia, which are also neural crest derived. At E11.5, *Sphk1* transcript was not detected in the sympathetic ganglia (Fig. 10). When we compared wild type, *Sphk1−/−;Sphk2+/−*, and *Sphk1−/−;Sphk2−/−* embryos, sympathetic ganglia appeared normal in all three genotypes (Fig. 10). This suggests that deleting *Sphks* did not impose non-specific defects in all periphery ganglia.

### Ablation of Sphk perturbed sphingolipid balance in the embryo

**Figure 11:**
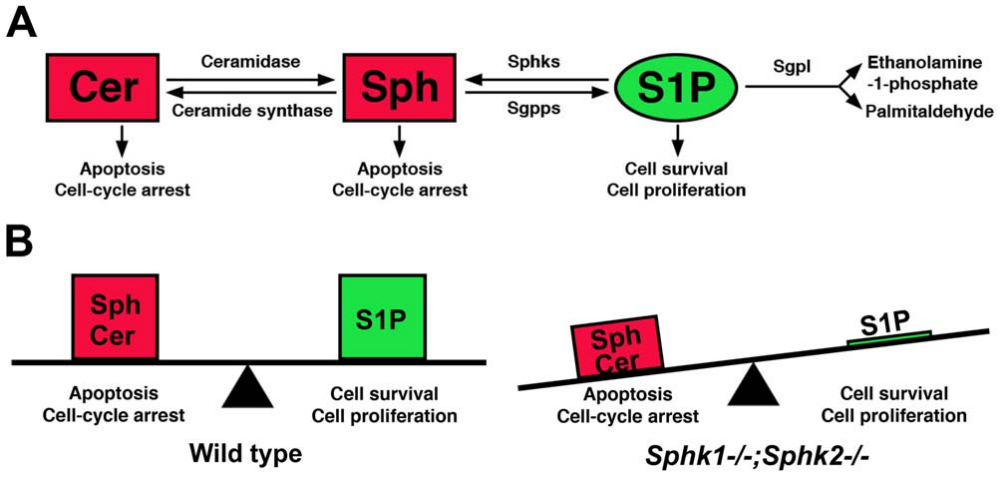
Schematic of S1P-SPHK1 and model of S1P-Sphingosine/ceramide balance. (**A**) A highly simplified schematic of S1P metabolic pathway. Sphingosine **(Sph)** is catalyzed by Sphingosine kinase 1 or 2 **(Sphks)** to form S1P. S1P can be degraded reversibly by Sphingosine phosphatase 1 or 2 **(Sgpps)** back to sphingosine or irreversibly by Sphingosine-1-phosphate lyase **(Sgpl)** into ethanolamine phosphate and hexadecenal. Sphingosine can be converted to ceramide **(Cer)** by Ceramide synthase, the reverse reaction is catalyzed by Ceramidase. Cumulative evidence from the literature suggests that S1P favors cell proliferation and promote survival while ceramide and sphingosine tend to drive cells into apoptosis and cell cycle arrest. (**B**) In wild type embryo where there is equilibrium of sphingosine/ceramide and S1P, cell death and survival are kept in balance. In the *Sphk1−/−;Sphk2−/−* double mutant, not only is there an overall decrease in sphingolipids including sphingosine and ceramide, S1P is completely absent. This results in a distortion of the sphingosine/ceramide to S1P ratio in the cells, which can tip the scale towards cell death and decrease in cell proliferation.

How does the absence of *Sphks* cause such catastrophic cell loss in the sensory ganglia? Sphingosine-1-phosphate (S1P) is formed by the phosphorylation of sphingosine by SPHK1 or SPHK2. Genetic deletion of both *Sphks* renders an animal with essentially no S1P [22], Table 2. One possible explanation for the cell death and proliferation phenotypes that we observe is that S1P usually stimulates cell growth; thus, its depletion can lead to a decrease in cell division. Moreover, with the accumulation of sphingosine due to the lack of SPHK activity, it is plausible that this substrate can be fed into the other end of the pathway and increases the level of ceramide, which favors cell death (Fig. 11). To test this idea, we collected wild type, *Sphk1−/−;Sphk2+/−*, and *Sphk1−/−;Sphk2−/−* embryos at E11.5 and measured their S1P and sphingolipid levels. As expected, there was little to no S1P in double mutant embryos that was above background level. In *Sphk1−/−;Sphk2+/−*, S1P level was reduced compared to wild type, which was also anticipated and had been reported previously [22], Table 2). To our surprise, sphingosine levels did not differ significantly in wild type, *Sphk1−/−;Sphk2+/−*, and *Sphk1−/−;Sphk2−/−* embryos, and ceramide level in *Sphk1−/−;Sphk2−/−* embryos was 50% and *Sphk1−/−;Sphk2+/−* 65% of wild type (Table 2).

**Table 2:** Sphingolipid measurements in control and *Sphk1−/−;Sphk2−/−* embryos.

pomole/sample	Wild type	*Sphk1−/−;Sphk2+/−*	*Sphk1−/−;Sphk2−/−*
S1P	5.90±1.00	1.39±0.14##	0.04±0.02** ††
Sph	19.79±2.44	18.92±3.00	13.77±2.70
ceramide	2164.14±200.28	1420.94±48.73#	1072.37±193.68**
**fold change compared to Wild type embryo**			
S1P	1.0000	0.2359	0.0062
Sph	1.0000	0.9564	0.6959
Ceramide	1.0000	0.6566	0.4955
**sphingolipid ratios**			
Ceramide/S1P	28.1923	78.4725	2,263.6738
Sph/S1P	3.3509	13.5862	377.8888

**p<0.01 *Sphk1−/−;Sphk2−/−* compare to Wild type.

†† p<0.01 *Sphk1−/−;Sphk2−/−* compare to Sphk1−/−; Sphk2+/−.

# p<0.05 *Sphk1−/−;Sphk2+/−* compare to Wild type.

## p<0.01 *Sphk1−/−;Sphk2+/−* compare to Wild type.

When we examined the data in more detail, we found that the sphingolipid levels were decreased across the board and S1P was absent in the double mutant. The balance of ceramide/S1P has been proposed as a “rheostat” to regulate cell growth and cell death [17], [18], [36]. Thus, we compared the relative ratios of ceramide/S1P and Sph/S1P in control and *Sphk1−/−;Sphk2−/−* animals. In wild type, the ratio of ceramide/S1P ratio was 28.2 and Sph/S1P was 3.4. In *Sphk1−/−;Sphk2+/−* embryos, ceramide/S1P and Sph/S1P ratios were elevated to 78.5 and 13.6, respectively. Because there was almost no S1P in the *Sphk1−/−;Sphk2−/−* double mutant, the ceramide to S1P ratio sharply increased to 2,263.70 and Sph/S1P to 377.9 (Table 2).

## Discussion

We have investigated the role of *Sphk1*, a key component in S1P signaling, in the developing sensory ganglia. We found that *Sphk1* was expressed first in neural crest progenitors then neurons in newly formed trigeminal and dorsal root ganglia. To investigate its function during development, we generated *Sphk1/Sphk2* double mutant mice that completely lacked *Sphk* activity. Absence of *Sphk*s leads to massive cell death in neurons and progenitors as well as near complete suppression of cell proliferation in the progenitor population. The *Sphk1* and *Sphk2* double knockout results showed that the phenotypes in the trigeminal and dorsal root ganglia are robust at E11.5—both neurons and progenitor cells are greatly depleted due to a combination of an increase in cell death and a decrease in cell proliferation. Upon examination of sphingolipid levels in the *Sphk* mutant, we saw an overall decline in multiple sphingolipids and essentially no S1P. Our data provide strong in vivo evidence that sphingosine kinase/S1P signaling has a novel role in regulating cell survival during development of sensory ganglia. Establishment of the sensory ganglia involves proper migration and condensation of neural crest stem cells, differentiation, and cell proliferation. In the *Sphk1/2* double mutant, we did not observe overt disruption of neural crest migration pattern, although the streams of cells appeared to be thinner. This suggests that depleting S1P in the embryo does not affect neural crest cell formation and migration per se but may affect their survival and/or proliferation.

Although the actions of S1P are usually transmitted through binding to its receptors, the sensory ganglia phenotype observed here could be explained at least in part, by SPHK activities. In contrast to the massive defects we observed in *Sphk1−/−;Sphk2−/−*, sensory ganglia from *S1pr1* and *S1pr2* mutant embryos are largely similar to their heterozygous litters and wild type controls. These data suggest that *Sphk1* and intracellular S1P signaling can play a role in sensory ganglia development.

Ceramide and sphingosine regulate stress response pathways and usually induced cell death and growth arrest. On the other hand, S1P tends to promote cell proliferation and inhibit cell death. Thus, the balance of intracellular ceramide, sphingosine, and S1P is important in regulating cell growth and survival [17], [18], [36]. In the *Sphk1/2* double mutant, there was essentially no S1P. Hence, we hypothesized that the massive decrease in cell survival of sensory ganglia lacking *Sphks* was caused by a large increase in ceramide and sphingosine in the absence of S1P. When we measured the levels of these sphingolipids, we found that there was no S1P in the double mutant as expected. There was also a four-fold decrease in S1P in *Sphk1−/−;Sphk2+/−* embryos compared to wild type, which is also expected. Ceramide level was decreased by 2-fold in *Sphk1−/−;Sphk2−/−* double knockout and there was a slight decrease in sphingosine. This was a bit surprising as we expected a buildup of sphingosine due to the absence of SPHK activity. We then considered the balance of these sphingolipids and sought to examine if they were altered. The ratio of ceramide:S1P was 28.2 in wild type and 78.5 in *Sphk1−/−;Sphk2+/−* embryos; however, this ratio rose dramatically to 2,263.7 in the double mutant due to the absence of S1P. The sphingosine/S1P ratio was 3.4 in wild type and 377.8 in *Sphk1−/−;Sphk2−/−* mutants, over a 100-fold increase. Therefore, although the pro-apoptotic ceramide and sphingosine were not increased, the lack of S1P disrupted the sphingolipid balance inside cells, favoring cell death and suppressing cell proliferation and survival. Our current study demonstrates that the relative ratios of these molecules are important in regulating cell survival in vivo.

During sensory ganglia development, several signaling pathways have been implicated in the survival and maintenance of neurons and precursor cells. For example, the family of neurotrophins and their receptors (Trk A, B, C) are involved in sensory neuron survival in both trigeminal and dorsal root ganglia [2], [5], [37]. It is unlikely that *Sphk1* is directly downstream of Trk receptors because it was clearly demonstrated that only neurons but not progenitor cells express Trk receptors in murine sensory ganglia [2] whereas *Sphk1* is expressed in both cell populations. It is possible that the p75 receptor pathway may indirectly influence *Sphk1* activity as it can activate ceramide and apoptotic pathways [38], [39], [40]. *Sphk1* is expressed by E10.5 in the sensory ganglia when p75 receptor can be observed; thus, it is plausible that *Sphk1* can act downstream of the p75 receptor directly or indirectly through ceramide. In addition to regulating cell death and proliferation in the developing nervous system, S1P/Sphingosine kinase signaling may modulate neuronal survival and functions in adult animals. For instance, in an in vitro model of brain ischemia, S1P was shown to reduce cell death, possibly via protein kinase C ε translocation to the mitochondria as well as reduction of Ca^2+^ in the mitochondria [41]. In adult hippocampal CA3 neurons, synaptic localization and activation of S1PR3, and generation of long-term potentiation were dependent on the production of S1P by SPHK1, as these activities were completely abolished in *Sphk1* null animals [42]. These data suggests that S1P/Sphingosine kinase signaling exert wide-ranging effects in the nervous system, some of which are receptor independent while others may require interactions with S1P receptors.

In summary, we have shown that the complete absence of Sphingosine kinase/S1P activity has a detrimental effect on early sensory gangliogenesis—assigning a novel role for sphingosine kinase in the developing nervous system. Our future goal is to identify signaling pathways that interact with S1P and mediate the proper formation of sensory ganglia.

## Supporting Information

Figure S1 **Sensory ganglia in** ***Sphk1−/−*** **and** ***Sphk2−/−*** **single knockout mice do not display any obvious defects.** Head and trunk transverse sections from E11.5 (40–42 somites) wild type, *Sphk1−/−* and *Sphk2−/−* mice were processed for Tuj1 **(red)** immunofluorescence and counterstained with DAPI **(blue)** to examine morphologies of the trigeminal **(Trig)** and dorsal root ganglia **(DRG)**. (**A,B**) Trunk sections showing E11.5 DRG in *Sphk1−/−* and *Sphk2−/−* embryos. DRG from *Sphk1−/−* (**A**) and *Sphk2−/−* (**B**) are similar in shape, size, and contain many Tuj1+ neurons; they look similar to wild type and *Sphk1−/−;Sphk2+/−* DRG in Fig. 3A–B. (**C,D**) Sections showing trigeminal ganglia from *Sphk1−/−* and *Sphk2−/−* embryos. Trigeminal ganglia from *Sphk1−/−* (**C**) and *Sphk2−/−* (**D**) are similar in shape, size, and contain many Tuj1+ neurons; they look similar to wild type and *Sphk1−/−;Sphk2+/−* trigeminal ganglia in Fig. 3D–E. **Scalebar** for A, B = 50 µm; **scalebar** for C,D = 100 µm.
(TIF)

Figure S2 ***Sphk2*** **expression in the developing sensory ganglia.** E10.5 (32–35 somites) and E11.5 (40–42 somites) transverse sections were processed for *Sphk2* (grey/black) section in situ hybridization followed by Tuj1 **(green)** and SOX10 **(red)** immunofluorescence. (**A,B**) E10.5 dorsal root ganglion **(DRG)**. (**C,D**) E10.5 trigeminal ganglion **(Trig)**. (**E,F**) E11.5 dorsal root ganglion **(DRG)**. (**G,H**) E11.5 trigeminal ganglion **(Trig)**. *Sphk2* is expressed at low to undetectable level in E10.5-11.5 dorsal root and trigeminal ganglia. **NT** = neural tube. **Scalebar** for A,B = 50 µm; **scalebar** for C,D = 50 µm; **scalebar** for E,F = 50 µm; **scalebar** for G,H = 50 µm.
(TIF)

Figure S3 **Apoptosis can be detected in neurons and progenitor cells in the** ***Sphk1−/−;Sphk2−/−*** **sensory ganglia.** E11.5 (40–42 somites) trigeminal and dorsal root ganglia from *Sphk1−/−;Sphk2−/−* embryos were immunostained with antibodies to SOX10 **(green; A, C, E)** and act-Casp-3 **(red)**, or islet-1/2 **(green; B, D, F)** and act-Casp-3 **(red)**. (**A,B**) Many act-Casp-3+ cells can be observed in E11.5 double mutant trigeminal ganglia. (**C**) High magnification images of boxed area in **A**. Some act-Casp-3+ **(red)** cells are closely associated with SOX10+ cells **(arrow)**. (**D** )High magnification images of boxed area in B. Some act-Casp-3+ **(red)** cells are also islet-1/2+ **(arrowhead)**. (**E,F**) Many act-Casp-3+ cells can be seen in E11.5 double mutant dorsal root ganglia. (**E**) Some act-Casp-3+ cells are closely associated with SOX10+ cells **(arrow)**. (**F**) We can also observe act-casp-3+ cells associated with islet-1/2+ nuclei **(arrowhead)**. **NT** = neural tube. **Scalebar** for A,B = 50 µm; **scalebar** for C,D = 50 µm; **scalebar** for E,F = 50 µm.
(TIF)
