# Supplementary figures and images for: Loss of Sphingosine Kinase 1/S1P Signaling Impairs Cell Growth and Survival of Neurons and Progenitor Cells in the Developing Sensory Ganglia
Source: PLoS One. 2011 Nov 9;6(11):e27150. doi: 10.1371/journal.pone.0027150 (PMC3212543; doi:10.1371/journal.pone.0027150)

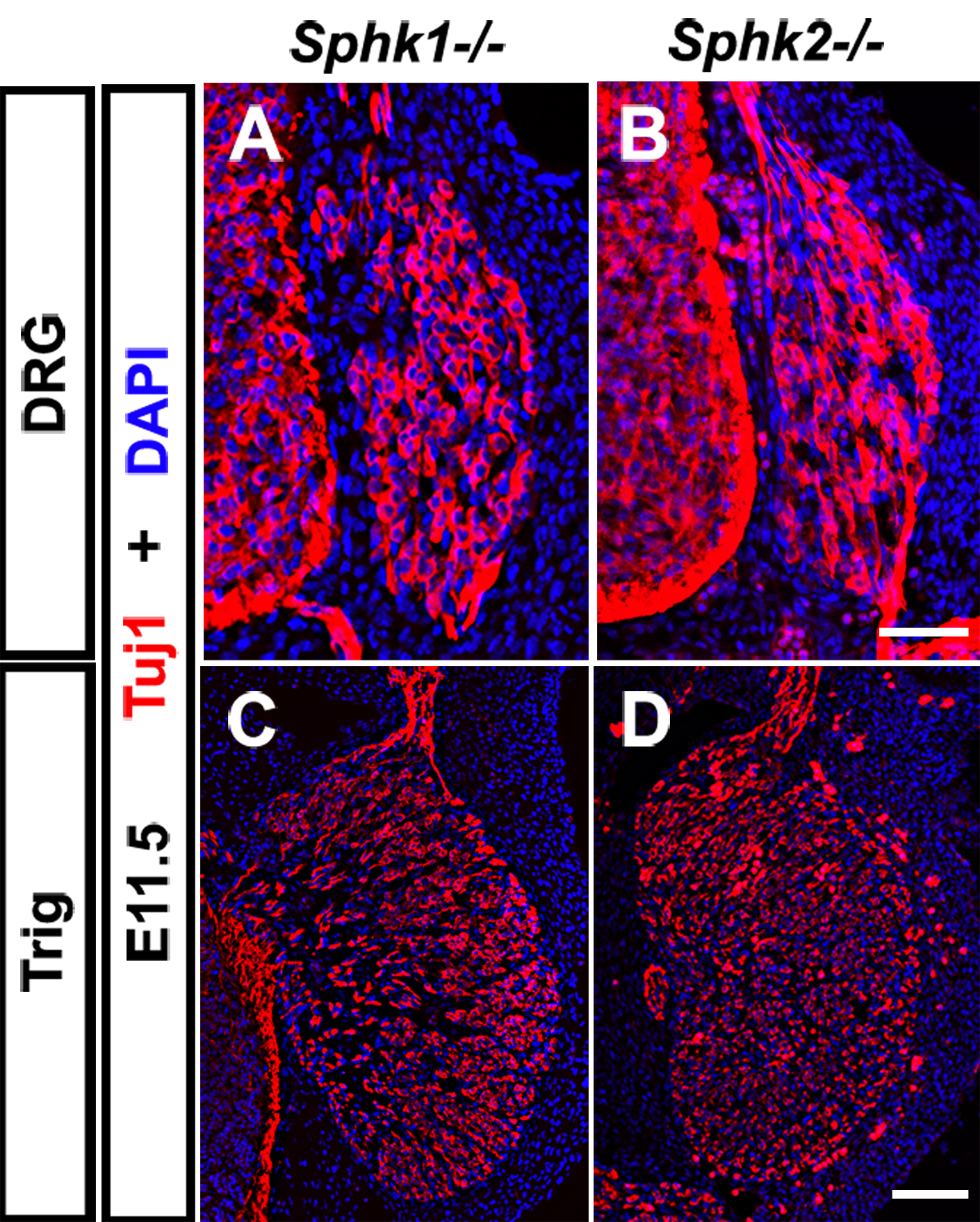

Supplement: Figure S1 — Sensory ganglia in Sphk1−/− and Sphk2−/− single knockout mice do not display any obvious defects. Head and trunk transverse sections from E11.5 (40–42 somites) wild type, Sphk1−/− and Sphk2−/− mice were processed for Tuj1 (red) immunofluorescence and counterstained with DAPI (blue) to examine morphologies of the trigeminal (Trig) and dorsal root ganglia (DRG). (A,B) Trunk sections showing E11.5 DRG in Sphk1−/− and Sphk2−/− embryos. DRG from Sphk1−/− (A) and Sphk2−/− (B) are similar in shape, size, and contain many Tuj1+ neurons; they look similar to wild type and Sphk1−/−;Sphk2+/− DRG in Fig. 3A–B. (C,D) Sections showing trigeminal ganglia from Sphk1−/− and Sphk2−/− embryos. Trigeminal ganglia from Sphk1−/− (C) and Sphk2−/− (D) are similar in shape, size, and contain many Tuj1+ neurons; they look similar to wild type and Sphk1−/−;Sphk2+/− trigeminal ganglia in Fig. 3D–E. Scalebar for A, B = 50 µm; scalebar for C,D = 100 µm. (TIF) [file pone.0027150.s001.tif]

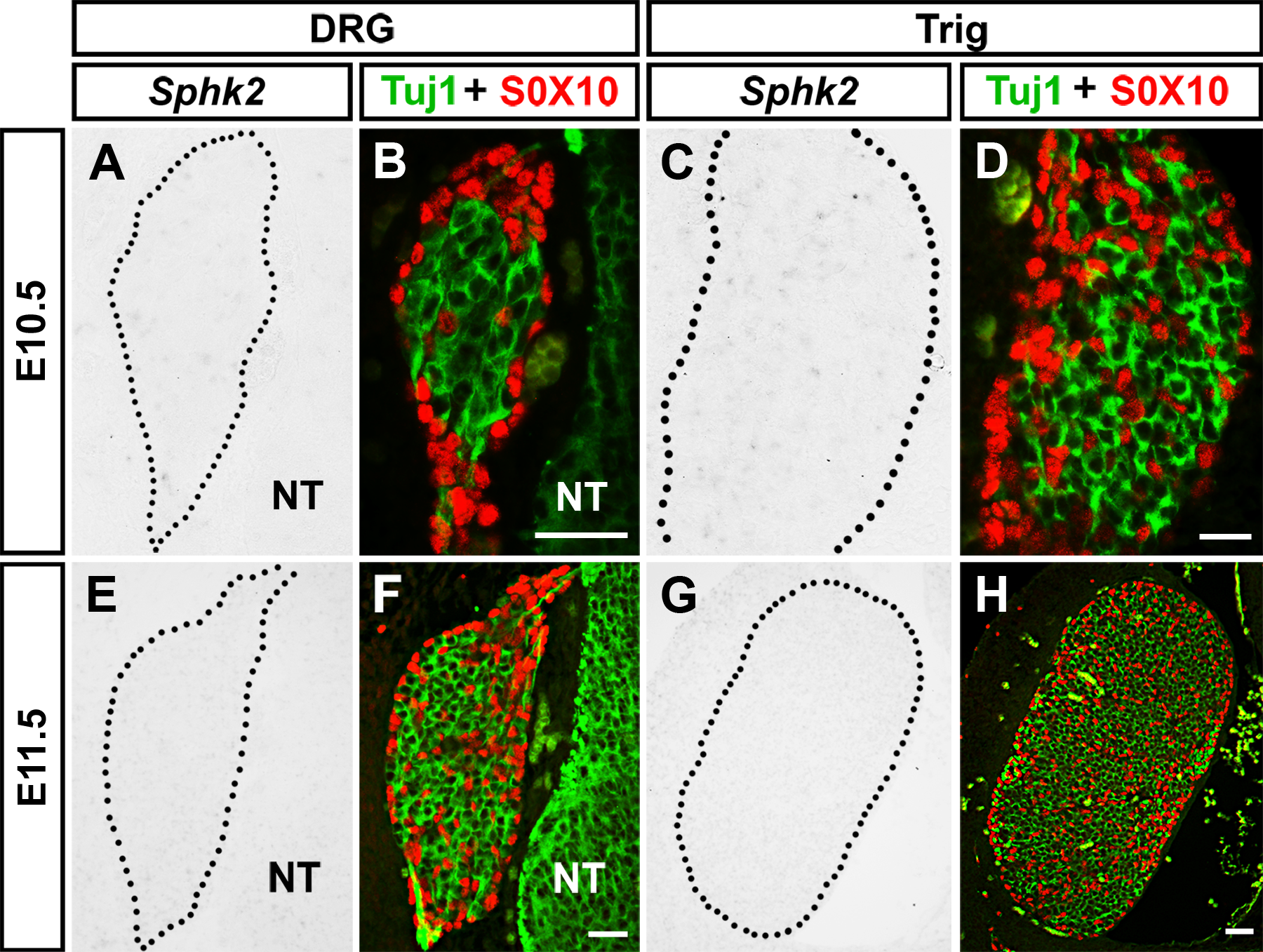

Supplement: Figure S2 — Sphk2 expression in the developing sensory ganglia. E10.5 (32–35 somites) and E11.5 (40–42 somites) transverse sections were processed for Sphk2 (grey/black) section in situ hybridization followed by Tuj1 (green) and SOX10 (red) immunofluorescence. (A,B) E10.5 dorsal root ganglion (DRG). (C,D) E10.5 trigeminal ganglion (Trig). (E,F) E11.5 dorsal root ganglion (DRG). (G,H) E11.5 trigeminal ganglion (Trig). Sphk2 is expressed at low to undetectable level in E10.5-11.5 dorsal root and trigeminal ganglia. NT = neural tube. Scalebar for A,B = 50 µm; scalebar for C,D = 50 µm; scalebar for E,F = 50 µm; scalebar for G,H = 50 µm. (TIF) [file pone.0027150.s002.tif]

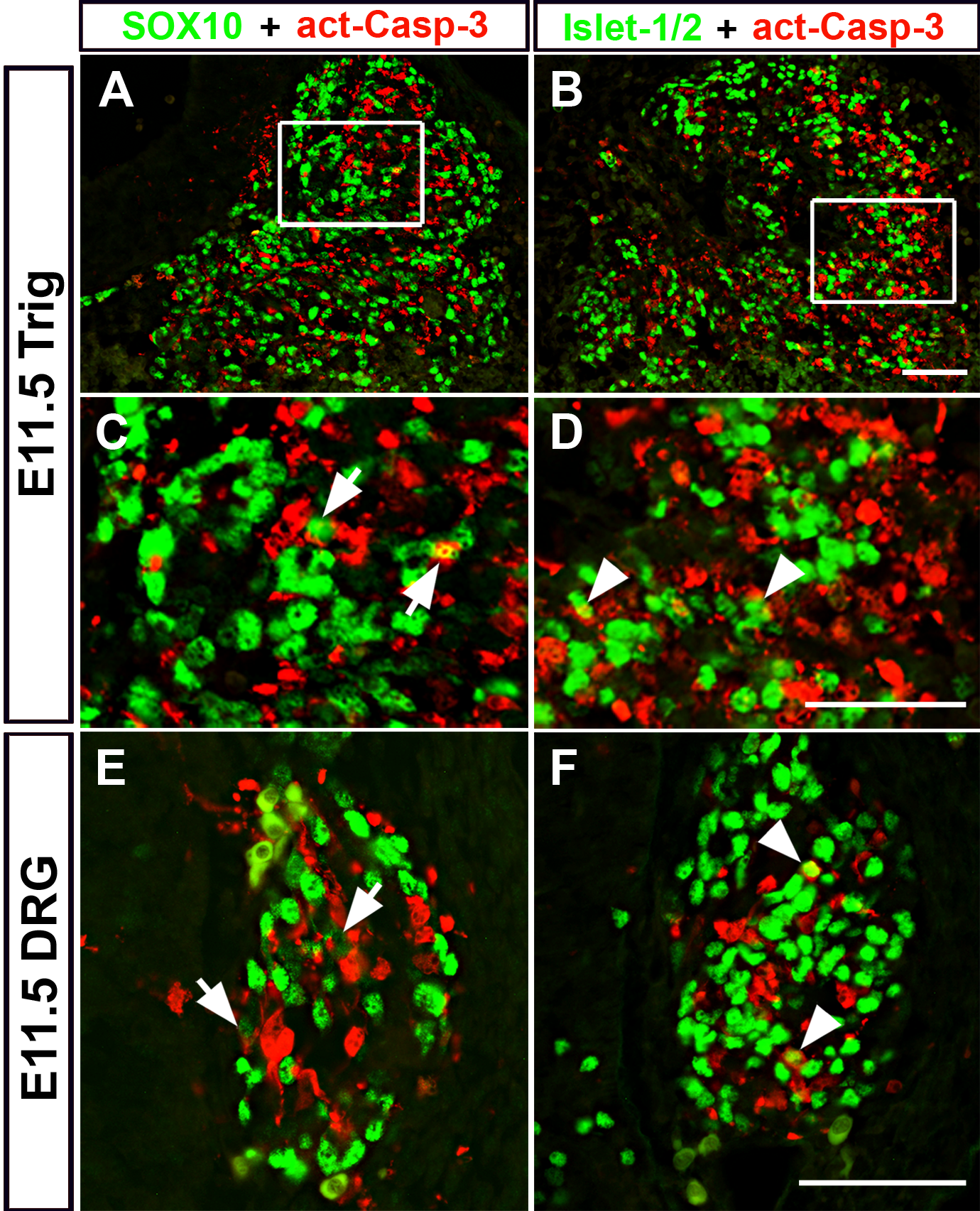

Supplement: Figure S3 — Apoptosis can be detected in neurons and progenitor cells in the Sphk1−/−;Sphk2−/− sensory ganglia. E11.5 (40–42 somites) trigeminal and dorsal root ganglia from Sphk1−/−;Sphk2−/− embryos were immunostained with antibodies to SOX10 (green; A, C, E) and act-Casp-3 (red), or islet-1/2 (green; B, D, F) and act-Casp-3 (red). (A,B) Many act-Casp-3+ cells can be observed in E11.5 double mutant trigeminal ganglia. (C) High magnification images of boxed area in A. Some act-Casp-3+ (red) cells are closely associated with SOX10+ cells (arrow). (D )High magnification images of boxed area in B. Some act-Casp-3+ (red) cells are also islet-1/2+ (arrowhead). (E,F) Many act-Casp-3+ cells can be seen in E11.5 double mutant dorsal root ganglia. (E) Some act-Casp-3+ cells are closely associated with SOX10+ cells (arrow). (F) We can also observe act-casp-3+ cells associated with islet-1/2+ nuclei (arrowhead). NT = neural tube. Scalebar for A,B = 50 µm; scalebar for C,D = 50 µm; scalebar for E,F = 50 µm. (TIF) [file pone.0027150.s003.tif]
